# Effect of the Application of Different Surface Treatment Methods on the Strength of Titanium Alloy Sheet Adhesive Lap Joints

**DOI:** 10.3390/ma12244173

**Published:** 2019-12-12

**Authors:** Anna Rudawska, Kazimierz Zaleski, Izabela Miturska, Agnieszka Skoczylas

**Affiliations:** Faculty of Mechanical Engineering, Lublin University of Technology, Nadbystrzycka 36 Str, 20-618 Lublin, Poland; k.zaleski@pollub.pl (K.Z.); a.skoczylas@pollub.pl (A.S.)

**Keywords:** titanium alloy, adhesive joint, strength, surface treatment, shot peening

## Abstract

This study investigated the effect of the different surface treatment methods on the strength of Ti6Al4V titanium alloy sheet adhesive joints. The following surface treatment methods were used: alkaline degreasing, anodizing, vibratory shot peening, and anodizing with vibrational shot peening. The following tests/measurements were carried out during the experiment: surface roughness measurements; microhardness measurements (conducted by the Vickers hardness test method); and strength tests of single-lap adhesive joints fabricated with the use of two epoxy adhesives, rigid and flexible, both based on epoxy resin Epidian 5. It has been found that the application of anodizing followed by vibratory shot peening leads to increased strength of adhesive joints, irrespective of the type of applied epoxy adhesive.

## 1. Introduction

The growing use of titanium and its alloys in the most demanding fields of technology results from its extremely attractive combination of strength and corrosion properties. Titanium and titanium alloys are used in the aircraft industry, aerospace industry, space technology, various medical applications, energy acquisition, and optics [1,2,3]. Elements made of titanium and its alloys used in these industries must very often be joined. It can be observed that titanium elements are more and more widely joined by adhesive bonding, a technique that constitutes an alternative to traditional joining methods [4,5,6,7,8,9,10]. Adhesive bonding is used for joining materials with specified properties, which are then used for making elements of various shapes located in various places of the structure. Adhesive bonding can be used to join elements of small sizes or complex shapes that are sometimes difficult to join using a different technology, because of, for example, lack of access or limited space in the structure. In adhesive bonding, one can distinguish many technological and constructional factors that affect the strength of fabricated adhesive joints. The technological factors include the following [3,11,12,13]: adherend surface preparation; adhesive type and the method of its application to the adherend surface; and adhesive layer curing conditions depending on, among others, adhesive type (temperature, time, and pressure) and seasoning conditions. Critical constructional factors include the following [3,14]: shape and dimensions of adhesive joints (which depend on, for example, the geometry of adherends), adhesive joint loading method. By making changes in the above factors, it can tailor properties of adhesive joints as desired.

Surface preparation is one of the first technological stages of the adhesive bonding process [3,4,11,13,15,16,17]. This stage is preceded by analysis of type and properties of the adherend, as these data greatly affect the selection of a surface preparation method along with agents and technological parameters applied in this operation. Surface preparation is the process of utmost importance owing to the necessity of producing a surface with required adhesive properties in order to obtain a high-strength adhesive joint. The selection of surface preparation methods in adhesive bonding depends on a number of factors. One of the most important factors concerning the selection of surface preparation techniques is the type of material. It has a significant impact on the selection of a surface preparation technique and its application. Surface preparation of titanium and titanium alloys for adhesive bonding usually involves the use of chemical treatment operations such as etching in sulfuric or nitric acid [4,18], anodizing [16,19], degreasing [18,20], and priming [14,21]. The latter is more frequently used, because in sulfuric acid etching, one can observe hydrogen adsorption by titanium. In addition to this, surface can also be prepared by plasma treatment [8,10,16,22]. Surface preparation of titanium and titanium alloys for adhesive bonding usually involves the use of mechanical treatment [8,15,18,23].

Review of surface pretreatments for titanium alloys especially with respect to their ability to improve the durability of bonded joints was presented by Critchlow and Brewis [12]. Pizzorini et al. [22] investigated the correlation between the surface characteristics of Ti–6Al–4V titanium alloy and the mechanical strength of the joints, preparing using epoxy adhesives. The results highlighted co-relationships between vacuum plasma surface treatment and mechanical resistance of the bonded joints. Mertens et al. [16] evaluated wet-chemical pre-treatments, like alkaline etching and anodising, and a plasma treatment for structural bonding of titanium Ti6Al4V. The results showed that atmospheric pressure plasma treatment of titanium is a promising technique to enhance the long-term adhesion on titanium. He at al. [19] presented the effects of anodizing temperature on the microstructure, composition, and surface profile of the oxide layers on Ti6Al4V and also on the shear strengths of the anodized alloy bonded with epoxy adhesive. The aim of research presented by Molitor and Young [20] was to evaluate alternative techniques for the bonding of a glass fibre reinforced composite structure to titanium alloy sheet metal component. Molitor et al. [4] also presented the review of the affect of several surface treatments on Ti6Al4V titanium alloy properties like: surface roughness, oxide layer, bond strength, and durability. Brack and Rider [10] examined the effect of mechanical (abrasion ad grit-blasting techniques) and chemical (air-plasma and organosilane) treatments on Ti6Al4V titanium alloy for bonding to rubber toughened epoxy adhesive. The effects of primer and annealing treatments on the shear strength between anodized Ti6Al4V and epoxy adhesive were investigated by He at al. [15]. Golaz et al. [18] investigated the different surface treatment of titanium (degreasing sandblasting, etching, anodizing) in various configurations in the context of ageing conditions and durability of bonded joints of Ti6Al4V titanium alloy. A combined surface treatment of sanding, degreasing, and chemical etching showed the best durability, whereas a treatment using an additional sulphuric anodic oxidation showed the best adhesion before ageing. Moreover, ageing decreased the bonded joints’ strength. 

Adhesively bonded machine components are often exposed to changing operational loads. One of the surface treatment methods that have a positive effect on fatigue life is shot peening, which consists of increasing the strength of a surface layer of material by bombarding it with a stream of metal shot. Shot peening is used for treating machine components made of titanium alloys [24,25,26,27,28]. The application of shot peening leads to increased fatigue life of a jet engine compressor disc made of titanium alloy. A positive effect of shot peening on the fatigue life of TB6 titanium alloy was reported by Yao et al. [25]. Increased fatigue life due to the application of vibratory shot peening was also observed for Ti6Al4V titanium alloy [26]. Wen et al. [28] investigated nano-crystallization on the surface layer of commercial pure titanium using high-energy shot peening.

The application of shot peening changes the surface layer properties of a treated element, predominantly its surface roughness, microhardness, and residual stresses. Increased fatigue life also greatly results from the occurrence of residual stresses in the surface layer of shot peened components. These compressive residual stresses are attributed to increased density dislocation, as confirmed by the positron annihilation tests conducted by Zaleski et al. [27]. Increased strength of the surface layer and residual stresses are associated with the free surface energy of shot peened elements. Previous studies have shown that the strength of adhesive lap joints depends on the employed adherend surface preparation method and its technological parameters [29]. 

This study investigates the effect of surface treatment methods on the strength of Ti6Al4V titanium alloy adhesive joints. Four surface preparation techniques are tested: alkaline degreasing, anodizing, anodizing combined with shot peening, and shot peening. After that, single-lap adhesive joints are fabricated with the use of epoxy adhesives.

## 2. Materials and Methods

The objective of the study was to determine the effect of surface preparation of Ti6Al4V titanium alloy specimens on the strength properties of adhesive lap joints fabricated with two different epoxy adhesive compositions: elastic and rigid. The study also included surface roughness and microhardness measurements of the surface layer of the adherends and determination of the shear strength of fabricated adhesive joints.

### 2.1. Adherend

Ti6Al4V titanium alloy (Titanium Grade 5) was used in the study. This alloy is widely used in different branches, including the air craft and aerospace industries and medicine. The mechanical properties of this alloy are shaped by heat treatment and plastic working. Heat treatment in particular affects the phase composition and microstructure of this alloy, and hence its mechanical properties. In the annealed state, this alloy has from 5% to 20% of the β phase. This alloy has good strength and plastic properties in the annealed state, and it can be shaped by heat treatment. The chemical composition and mechanical properties of Ti6Al4V alloy according to ISO 5832 standard are given in Table 1 and Table 2.

**Figure 1:**
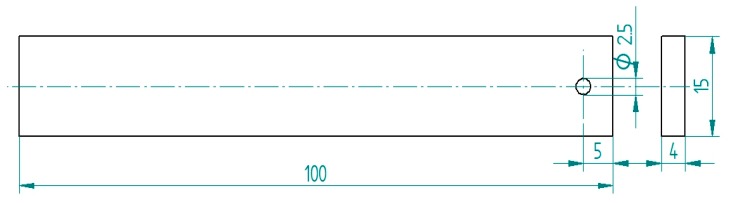
Geometry and dimensions of the adherend.

Test specimens were cut out from one metal sheet of 4 mm in thickness. Dimensions of the prepared specimens are given in Figure 1. Cut-out specimens had their sharp edges made blunt, while their surface was ground with abrasive paper. Finally, technological holes of 2.5 mm in diameter were made.

### 2.2. Surface Preparation Methods

Four surface preparation methods used in the tests are listed in Table 3.

Surface preparation for anodizing included the following steps: (i) alkaline degreasing, (ii) cascade rinsing in cold deionized water, (iii) acid etching, and (iv) rerinsing. All of these operations were carried out before anodizing for some part samples. However, some of the samples were given only with alkaline degreasing. The first step, alkaline degreasing, was performed in a mixture of sodium tripolyphosphate (Na_5_P_3_O_10_), sodium tetraborate (Na_2_B_4_O_7_), and deionized water by immersion bath. The solution had a temperature of 55 °C and an acidity of 8 pH. The alkaline degreasing was performed for 55 min. The solution in the bath was constantly mixed during the degreasing operation. Cascade rinsing, the second step for the preparation of samples, was performed in ambient temperature for 2 min in order to clean the surface after alkaline degreasing. To remove produced oxides, the acid etching operation (iii) was performed in a mixture of hydrofluoric acid (HF) and nitrogen acid (HNO_3_) at ambient temperature for 30 min. Rerinsing, the last step before anodizing, was performed in deionized water at an ambient temperature for 5 min. The anodizing operation, after the preparation stage, was performed in sulfuric acid with a current density of 0.5 A/dm^3^ for 15 min. After anodizing, the specimens were rinsed in cold deionized water at an ambient temperature for 2 min and dried in compressed deoiled and dehydrated air. After that, some anodized and non-anodized specimens were subjected to vibratory shot peening that consisted of impacting the surface of specimens fixed in the vibrator work chamber with a stream of metal shot [26]. The shot peening process, based on the preliminary studies [30], was described with the following parameters: vibration amplitude a = 60 mm, vibration frequency ν = 7 Hz, shot diameter d = 6 mm, and time t = 10 min. Shot elements were made on the steel 100Cr6 with hardness 60 HRC. The degree of the cover was 100% and the resulting intensity was 0.4 mmA.

### 2.3. Shape and Dimensions of Adhesive Joints and Specimen Preparation Conditions

Ti6Al4V titanium alloy sheet adhesive joints were fabricated with the use of two types of epoxy adhesives; their chemical compositions are given in Table 4.

**Figure 2:**
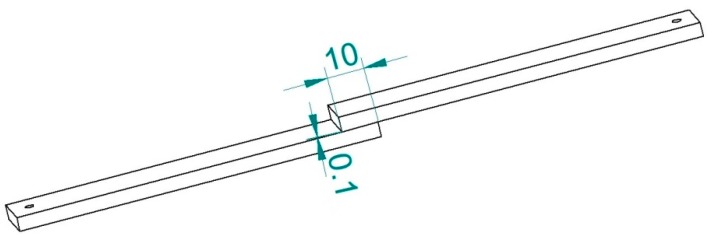
Schematic of adhesive joints used in tests (dimensions in mm).

The first epoxy adhesive contains: Epidian 5 epoxy resin and polyamide curing agent—polyaminoamide C (trade name—PAC). The second epoxy adhesive consists of Epidian 5 epoxy resin and amine curing agent—triethylenetetramine (trade name—Z-1). Epidian 5 epoxy resin is unmodified epoxy resin based on bisphenol A. All epoxy adhesive components were manufactured by CIECH Sarzyna S.A, Poland (http://www.zch.sarzyna.pl). The properties of epoxy adhesives components (resins and curing agents) as well as the mechanical properties of cured epoxy adhesives were described in the works of [31,32]. The resin and curing agent were mixed with the use of a mechanical mixer provided with a dispersion disk. The application of this type of mixer ensures a high degree of homogenization between the resin and the curing agent. Adhesive composition ingredients were mixed for 2 min with the shear rate of 128 m/min and then degassed for 2 min. The adhesive compositions were prepared directly before use. Each adhesive was hand-applied with a pipette to one of the adherend surfaces to ensure the same dosage of the adhesive and thus to obtain adhesive layers with the same thickness of g_k_ = 0.1 mm. Lap length was (approximately) set to be equal to 10 mm. A schematic of the fabricated adhesive joints is given in Figure 2.

Technological holes made in the specimens of the adhesively bonded material ensured axiality and alignment of the adherends. Adhesive layers were left to cure for seven days. The single-stage curing process was performed at an ambient temperature (22 ± 1 °C), with an air humidity range of 32% ± 2%. To curing process was performed under a pressure of 0.1 MPa. Four batches of adhesive joints were fabricated (in accordance with four types of surface preparation—Table 3), with each batch containing eight adhesive joints.

### 2.4. Surface Roughness and Microhardness Measurements

Prior to adhesive bonding, surface roughness and topography of the prepared Ti6Al4V alloy sheets were measured with Hommel-Etamic’s T8000 RC 120–140 device, in compliance with PN-EN ISO 13565-2 standard. The device also allows surface contour measurements. The sampling length was set at lr = 0.8 mm. The following surface roughness profile parameters were analyzed: Ra—roughness average, Rsk—profile asymmetry (in older standards known as “skewness”), Rt—maximum height of the profile, and Rp—maximum profile peak height. Seven measurements were taken per each specimen. 

On the basis of the obtained results, an index of the decrease in surface roughness K_R_ was determined:

(1) $$K_{R}=\frac{R^{\prime }}{R},$$

where, $R^{\prime }$—roughness parameter before shot peening; R—roughness parameter after shot peening.

Microhardness was measured on specimen surface by Vickers hardness test method in compliance with PN-EN ISO 6507. Measurements were made with Leco’s LM 700at (Leco, San Jose, MI, USA) microindentation hardness tester. Two indentation loads were applied: 100 g (HV 0.1) and 300 g (HV 0.3).

### 2.5. Strength Test

The strength tests of shear-loaded titanium alloy sheet adhesive joints were performed on the Zwick/Roell 150 testing machine (Zwick/Roell, Wroclaw, Poland) in compliance with PN-EN 1465 standard to determine their failure load and shear strength. The traverse displacement velocity was set to be equal to 5 mm/min at a preload of 5 N. After curing, the specimens were fixed in screw-wedge fixtures of the testing machine.

## 3. Results

### 3.1. Surface Roughness and Microhardness Results

**Figure 3:**
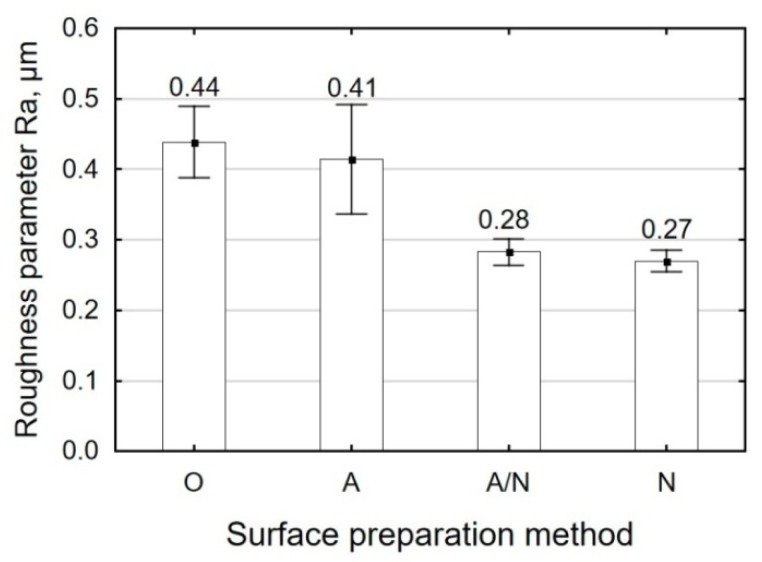
Surface preparation method versus arithmetic average of profile roughness.

Figure 3 shows the effect of surface preparation technique on the roughness parameter Ra. The results demonstrate that the arithmetic average of the ordinates obtained for the surfaces after alkaline degreasing (O) and that obtained for the anodized surface (A) are similar and amount to Ra ≈ 0.42 µm. After shot peening (N), the surface roughness parameter Ra decreased to approximately 0.27 µm, that is, by 35% compared with its pre-treatment value, irrespective of the employed surface preparation method.

**Figure 4:**
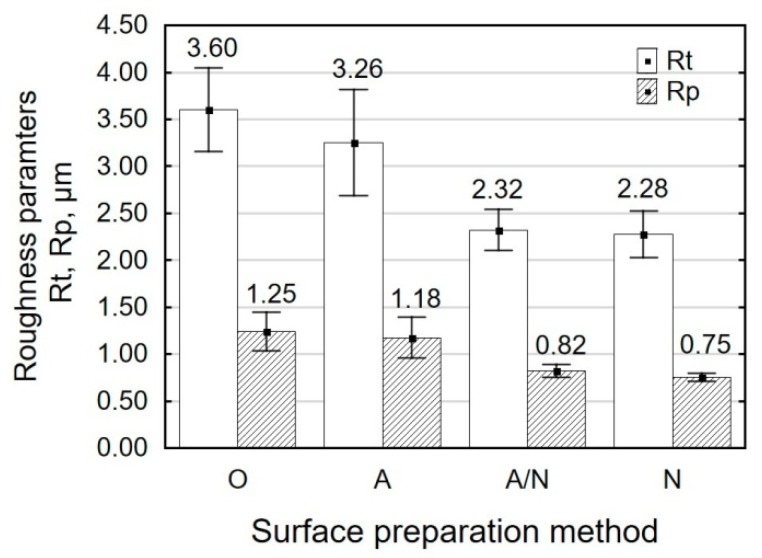
Surface preparation method versus profile height parameters (Rt, Rp).

Examining the profile height parameters Rt and Rp (Figure 4), it can be observed that—similarly to Ra—these parameters for the surfaces treated by alkaline degreasing (O) and anodizing (A) are on a similar level. As a result of regular impact of metal shot on the treated surface, surface microirregularities get beveled and flattened. The profile roughness decrease parameter K_Rt_ for the shot peened surface (N) is 1.58, while for the surface treated by anodizing and shot peening (A/N), it is equal to 1.41. The energy generated by metal shot leads to the deformation of microirregularity peaks and, as a result, the maximum profile peak height Rp decreases by 40% for the specimens treated by vibratory shot peening (N) and by 39% for the specimens treated by anodizing and shot peening (A/N).

**Figure 5:**
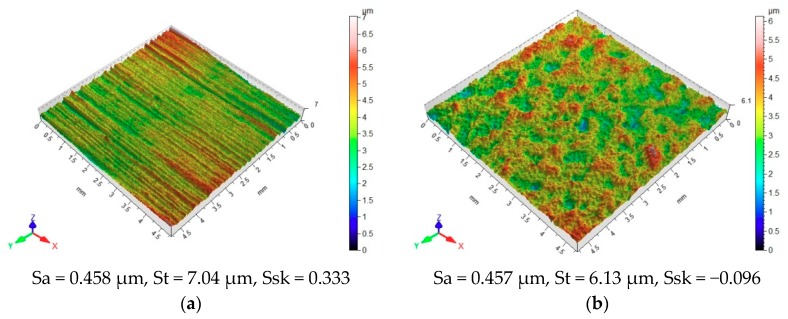
Preparation method versus surface topography after (**a**) anodizing (A); (**b**) anodizing and vibratory shot peening (A/N).

Vibratory shot peening has an impact on the geometric structure orientation of the assessed surface (Figure 5). The surface before shot peening, but after anodizing (A) (Figure 5a) exhibits a parallel distribution of irregularities, which were formed as a result of grinding with abrasive paper.

**Figure 6:**
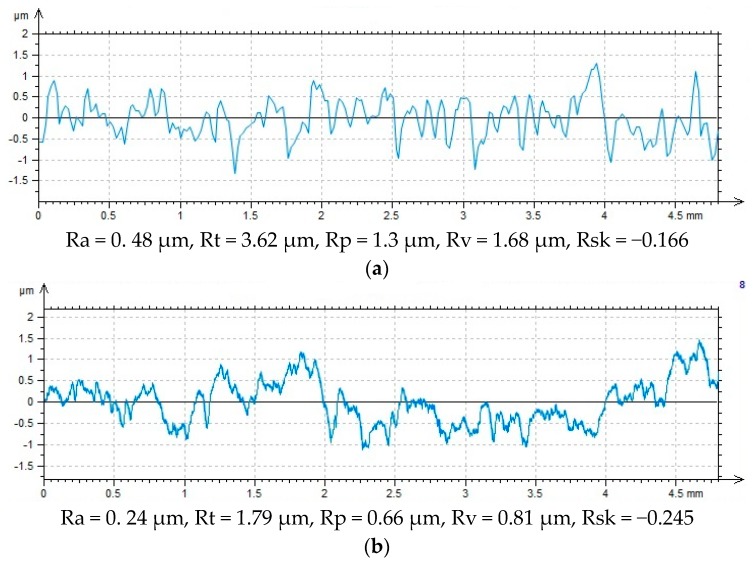
Surface profilograms after (**a**) anodizing (A); (**b**) anodizing and vibratory shot peening (A/N).

One can notice steep peaks and valleys, as proven by the profilogram of the surface before treatment (Figure 6a) and the high value of St (total profile roughness height). Shot peening produces peaks and cavities with a smaller height and rounded peaks and valleys (Figure 6b). 

**Figure 7:**
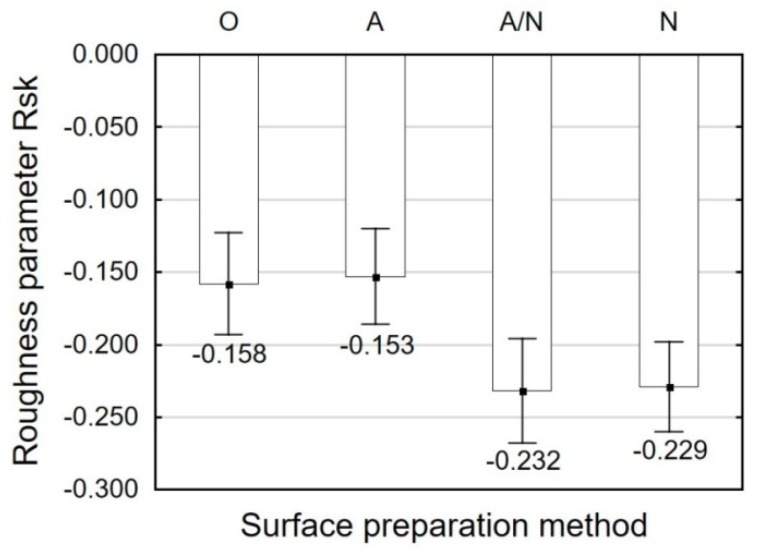
Surface preparation method versus profile roughness asymmetry Rsk.

Thereby, the shaped surface can have high adhesive properties, as confirmed by the profile roughness asymmetry parameter Rsk assessed in a 2D system (Figure 7) and the skewness of topography height distribution Ssk. The application of shot peening leads to an increase in the absolute value of Rsk. This means that this surface material is characterized by material concentrated in the vicinity of profile peaks, and hence it has a shape of plateau with high retention of a lubricating agent or adhesive.

The results plotted in Figure 6 and Figure 7 indicate high discrepancies between the parameters Ssk and Rsk. The Ssk parameter describes the surface with an area of 4.8 mm × 4.8 mm, while the Rsk parameter is a measurement average calculated on a measuring length of 4.8 mm. The 4.8 mm × 4.8 mm surface has numerous peaks and valleys that counterbalance on the analyzed area. 

**Figure 8:**
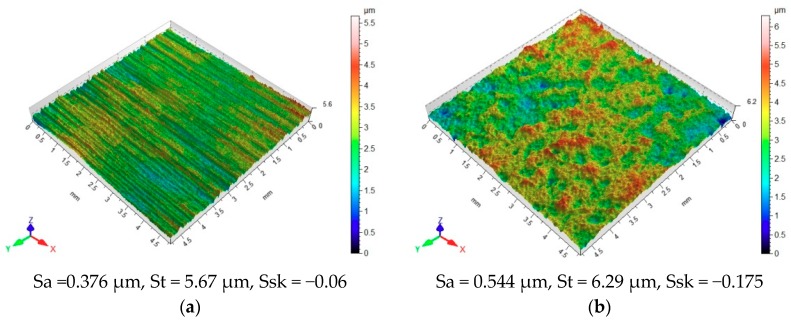
Preparation method versus surface topography after (**a**) degreasing (O); (**b**) vibratory shot peening (N).

**Figure 9:**
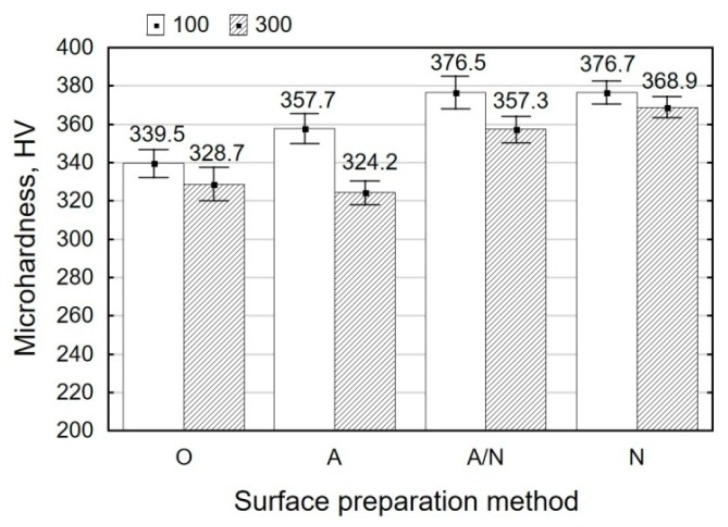
Surface preparation method versus microhardness.

Comparing the surface topographies after degreasing (O) and vibratory shot peening (N), it can be observed that there are no significant differences in the amount of resulting micro-unevenness, as shown in Figure 8. Figure 9 illustrates the effect of surface preparation methods on obtained microhardness.

The use of anodizing (A) led to a slight increase in hardness by 5.4% when compared with the results obtained for the alkaline degreased surface (O), as shown by the measurements under a load of 100 g. The produced hardened layer has a small thickness. This is confirmed by the results of the microindentation hardness tests conducted under a load of 300 g, where the obtained microhardness is similar to that describing the specimens only treated by alkaline degreasing (O). The use of vibratory shot peening led to a slight increase in the degree of strain hardening of the treated surface. The degree of strain hardening of the shot peened specimens (N) is 10.9%, while that obtained for the specimens treated by anodizing and vibratory shot peening (A/N) amounts to 4.97%.

### 3.2. Strength Test Results

**Figure 10:**
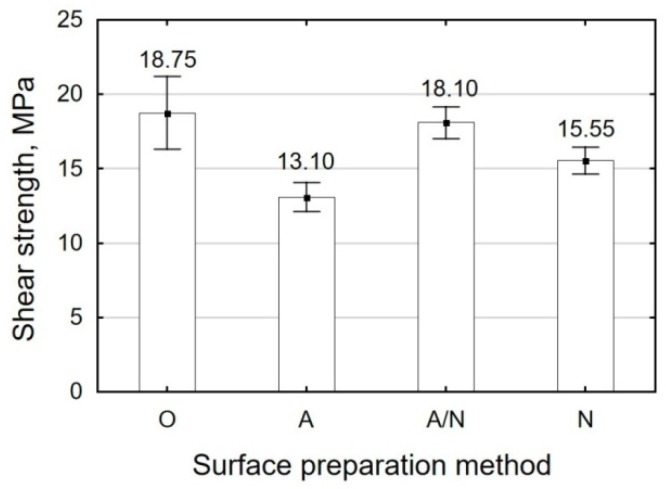
Shear strength of Ti6Al4V alloy sheet adhesive joints fabricated with E5/PAC/100:80.

**Figure 11:**
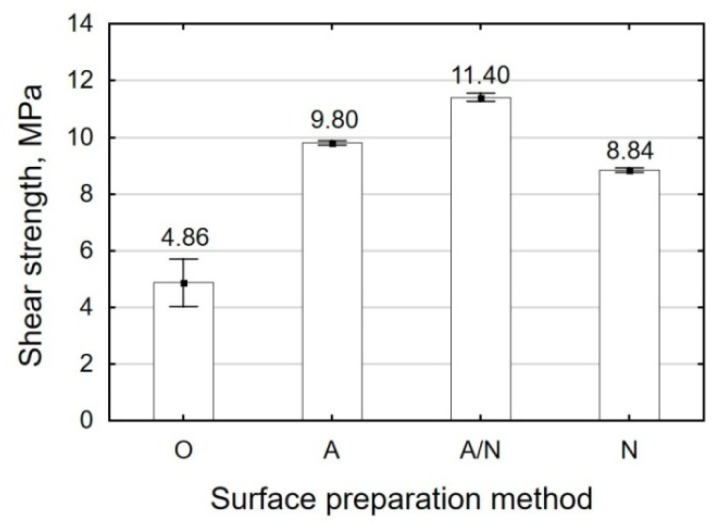
Shear strength of Ti6Al4V alloy sheet adhesive joints fabricated with E5/Z-1/100:10.

The results of the strength tests for Ti6Al4V titanium alloy sheet adhesive joints are given in Table 5 and in Figure 10 and Figure 11.

The results (Figure 10) obtained for the Ti6Al4V alloy sheet adhesive joints fabricated with the adhesive composition containing Epidian 5 and curing agent PAC (E5/PAC/100:80) demonstrate the following:

- The highest shear strength can be observed for the specimens treated by alkaline degreasing (O) (18.75 MPa) and for the specimens treated by anodizing followed by vibratory shot peening (A/N) (18.10 MPa);
- The shear strength of adhesive joints where surface preparation treatment involved the application of vibratory shot peening after anodizing (A/N) is higher by 28% than the shear strength of the specimens that were only treated by anodizing (A) and by 14% than the shear strength of the adhesive joints where the surface was prepared by vibratory shot peening (N);
- The shear strength of the specimens treated by vibratory shot peening (N) is higher than that the specimens treated by anodizing (A). The difference in adhesive joint strength between these surface preparation variants is 16%.

Considering the results given in Figure 11, one can make the following observations with respect to the adhesive joints fabricated with the E5/Z-1/100:10 adhesive composition:

- The lowest shear strength is obtained for the adhesive joints where the surface preparation treatment involved alkaline degreasing (O) and is equal to 4.87 MPa, which amounts to 57% of the maximum strength (11.40 MPa) obtained for the adhesive joints in which the surface was treated by anodizing followed by vibratory shot peening (A/N);
- Like in the case of titanium alloy sheet adhesive joints fabricated with E5/PAC/100:80, the shear strength of the adhesive joints in which surface preparation involved the use of anodizing and vibratory shot peening (A/N) is higher than the shear strength of the adhesive joints in which surface treatment involved anodizing (A) and shot peening (N) by 14% and 22%, respectively.

The differences in the obtained shear strength results are also caused by the use of two different epoxy adhesives. The PAC-cured epoxy adhesives exhibit a higher elasticity than the epoxy adhesives cured with Z-1. For every considered surface preparation method, the titanium alloy sheet adhesive joints prepared with E5/PAC/100:80 have higher shear strength than the adhesive joints fabricated with E5/Z1/100:10. Depending on the applied surface preparation method, these differences were as follows: O—74%, A—25%, A/N—37%, N—43%. Irrespective of the applied epoxy adhesive, both types of adhesive joints have higher shear strength when anodizing is followed by vibratory shot peening. It can be observed that the application of this combined surface treatment variant A/N produces higher-strength adhesive joints than when the two operations are applied separately.

## 4. Discussion

A comparison of obtained shear strength results and surface roughness profile parameters demonstrates that the use of shot peening brings desired results for the adhesive joints fabricated with E5/Z-1/100:10. Shot peening leads to the formation of numerous cavities on the surface of both anodized and non-anodized specimens; these cavities are potential surface irregularities penetrated by adhesives. This is confirmed by the obtained values of the skewness parameter Rsk, which for the shot peened surface is higher by 44% for the non-anodized specimens and by 51% for the anodized specimens, when compared with the specimens subjected to alkaline degreasing. Ingram and Ramani [33] underlined, among other things, that the higher initial roughness of titanium alloy surface after anodisation promotes initial mechanical interlocking and initial strength.

On an extensive topography surface, there is a natural "mesh" of the adhesive with the surface of the material because adherent in liquid form before curing fills unevenness on the surface of the adherend. However, not always does the more developed surface work in favor of the joints. The adhesive viscosity has a great influence on mechanical adhesion. A highly viscous substance will not fills mall, numerous surface irregularities that will be created on a higher surface roughness. Owing to the presence of cavities with rounded valleys and beveled peaks, surface irregularities can be more thoroughly filled with E5/Z-1/100:10 adhesive compositions, as its combination with the Z-1 curing agent with a viscosity ranging from 20 to 30 mPa·s at 25 °C produces a composition with decreased viscosity and density ranging from 0.978 to 0.983 g/cm^3^ at 20 °C.As for E5/PAC/100:80 adhesive composition, the specimens subjected to alkaline degreasing (O) have a higher shear strength than those whose surface was prepared by vibratory shot peening (N). This probably results from the surface geometry characteristics. The unidirectional surface micro-irregularities with sharp peaks and valleys ensure better “filling” of the adherend surface with E5/PAC/100:80adhesive compositions. The combination of Epidian 5 with PAC—its viscosity at 25 °C ranging from 10,000 to 25,000 mPa·s and density at 20 °C of 1.10–1.20 g/cm^3^—after curing produces a more elastic adhesive layer than the adhesive layer created by E5/Z-1/100:10 adhesive compositions. 

These results, taking into account the type of adhesive, also confirm the results of research received by the authors in the work of [34]. Rudawska [34] emphasized that the use of more flexible adhesive in the case of a rough surface leads to greater adhesive joint strength (of aluminium alloys) than the use of more rigid adhesive. According to da Silva and Lopes [35], an ideal adhesive joints is one in which the adhesive flexibility and strength properties vary along the overlap length, and they investigated the mixed-adhesive technique (various type of adhesive for preparing the adhesive joints). Da Silva et al. [36] investigated the single lap joints with patterns with and without chemical treatment and bonded a brittle and ductile adhesive. They underlined that (among other things) the patterns can increase the joints’ strength of non-treated adherends in the case of the brittle adhesive.

As for the anodized specimens, the shear strength is higher for the adhesive joints where vibratory shot peening was used for adherend surface preparation. This can probably be attributed to the surface layer energy properties that are produced owing to the use of anodizing followed by vibratory shot peening.

## 5. Conclusions

On the basis of the strength results obtained for of Ti6Al4V titanium alloy sheet adhesive joints, one can reach the following conclusions:

- The use of a hybrid surface preparation treatment, that is, anodizing combined with vibratory shot peening, results in increased strength of Ti6Al4V alloy sheet adhesive joints; in contrast, when these two surface preparation techniques are used separately, the strength of the produced adhesive joints is lower;
- To produce higher-strength Ti6Al4V alloy sheet adhesive, it is recommended to use a more flexible adhesive;
- Both the properties of an adhesive (particularly its viscosity) and the geometric structure of adherend surface, after the application of specified surface treatment methods, significantly affect the production of a real adhesive–adherend contact surface, as this ensures relatively high strength because of, among others, a considerable role of mechanical adhesion;
- The increase in the ratio between valley depth and peak height due to the application of shot peening, as shown by the decrease in the Rsk parameter, has a positive effect on the strength of adhesive joints;
- Increased curing of the adherend surface layer due to the application of shot peening can lead to increased strength of adhesive joints loaded by variable forces.

## Figures and Tables

**Table 1:** Chemical composition of Ti6Al4V titanium alloy (according to PN-EN ISO 5832-3).

Titanium Alloy	Chemical Composition, %							
	Al	V	Fe	O	H	C	N	Ti
Ti6Al4V	5.5	3.5	<0.30	<0.20	<0.0015	<0.08	<0.05	Other

**Table 2:** Mechanical properties of titanium alloys (according to PN-EN ISO 5832-3).

Mechanical Properties	Value	Unit
Young’s Modulus	110–114	GPa
Tensile Strength R_m_	960–970	MPa
Yield Point R_p0.2_	850–900	MPa

**Table 3:** Description of surface preparation methods for Ti6Al4V alloy specimens.

Surface Preparation Method	Denotation
Alkaline Degreasing	O
Anodizing	A
Anodizing and Vibratory Shot Peening	A/N
Shot Peening	N

**Table 4:** Epoxy adhesive compositions used in tests.

Epoxy Resin	Curing Agent	Stoichiometric Ratio Epoxy Resin: Curing Agent	Denotation
Epidian 5	Polyamide Curing Agent (PAC)	100:80	E5/PAC/100:80
Epidian 5	Amine Curing Agent (Z-1)	100:10	E5/Z1/100:10

**Table 5:** Failure force of Ti6Al4V alloy sheet adhesive joints.

Adhesive Composition	Surface Treatment	Failure Force	
		Mean, N	Standard Deviation, N
E5/PAC/100:80	O	3040	495
	A	1960	14
	A/N	2625	276
	N	2270	71
E5/Z1/100:10	O	754	161
	A	1700	212
	A/N	1700	127
	N	1340	57
